# *Bifidobacterium animalis* subsp. *lactis* HN019 Effects on Gut Health: A Review

**DOI:** 10.3389/fnut.2021.790561

**Published:** 2021-12-14

**Authors:** Jing Cheng, Arja Laitila, Arthur C. Ouwehand

**Affiliations:** International Flavors & Fragrances Inc., Global Health and Nutrition Science, Danisco Sweeteners Oy, Kantvik, Finland

**Keywords:** bowel function, diarrhea, gut motility, *Bifidobacterium animalis* subsp. *lactis* HN019, intestinal barrier, probiotic, gut–brain

## Abstract

Optimal gut motility is central to bowel function and gut health. The link between the gut dysmotility related disorders and dysfunctional-intestinal barriers has led to a hypothesis that certain probiotics could help in normalizing gut motility and maintain gut health. This review investigates the roles of *Bifidobacterium animalis* subsp. *lactis* HN019 (*B. lactis* HN019™) on gut health, and its mechanisms of action in various pre-clinical and clinical studies. Research supports the hypothesis that *B. lactis* HN019™ has a beneficial role in maintaining intestinal barrier function during gastrointestinal infections by competing and excluding potential pathogens via different mechanisms; maintaining normal tight junction function *in vitro;* and regulating host immune defense toward pathogens in both *in vitro* and human studies. This has been observed to lead to reduced incidence of diarrhea. Interestingly, *B. lactis* HN019™ also supports normal physiological function in immunosenescent elderly and competes and excludes potential pathogens. Furthermore, *B. lactis* HN019™ reduced intestinal transit time and increased bowel movement frequency in functional constipation, potentially by modulating gut–brain–microbiota axis, mainly via serotonin signaling pathway, through short chain fatty acids derived from microbial fermentation. *B. lactis* HN019™ is thus a probiotic that can contribute to relieving gut dysmotility related disorders.

## Introduction

Bowel function plays a central role in gut health and overall well-being. A healthy gut involves many factors, including intact epithelial barrier function, homeostatic intestinal microbiota, optimal functioning digestive organs (stomach, liver/gallbladder, pancreas), and definitely optimal gut motility. The interactions of those systems are in homeostasis in healthy subjects with normal bowel function and balanced immune function. However, this can be perturbed by antibiotic usage, unbalanced diet, and other life-style factors, infections, and other disease conditions. This may lead to changes in bowel habits and stool consistency, diarrhea, constipation, or a spectrum of both them, such as manifested in different subtypes of irritable bowel syndrome (IBS) (1–3).

The human gastrointestinal tract (GIT) represents an extremely complex ecosystem, comprising of interactions within the digestive system as well as cross-functioning with various immune cells, endocrine cells, and nerve cells, and organ systems beyond the GIT (4–6). Evidence also showed that the interactions between GIT and other organs often involve the residential intestinal microbiota, which is a dense community (estimated to 4 × 10^13^ microbial cells), contributing to various metabolic functions, and immunological defenses in the maintenance of normal health (7–9). Therefore, gut health has traditionally been one of the most important targets for the majority of probiotic applications, and central for connections to other health benefits via the intestinal microbiota.

Probiotics are defined as live microorganisms that, when administered in adequate amounts, confer a health benefit on the host (10). Studies have shown that probiotics are beneficial for supporting the human body's natural functions in both health and disease; either by interacting with the host directly or indirectly by optimizing the composition and/or activity of the intestinal microbiota. Although common probiotic benefits have been proposed, the core benefits are strain-specific (10).

*Bifidobacterium animalis* subsp. *lactis* HN019™ (*B. lactis* HN019™) is a strain originally isolated from commercial yogurt, and commercialized as an ingredient for dietary supplements, fermented and non-fermented foods, and beverages for decades (11). Its complete genome sequence was published in 2018 (12), allowing for stringent control of product quality, safety, purity, and consistency by strain identity confirmation at industrial scale. *B. lactis* HN019™ is available in various finished formats, granting it profound application potential in food and beverage, dietary supplement, and pharmaceutical industries.

The objective of this review is to discuss the properties of *B. lactis* HN019™ in the context of both preclinical and clinical studies on the following aspects of gut health; survival through the gut, modulation of intestinal microbiota, maintain intestinal barrier functions during gastrointestinal infections, regulate gut motility, and improve symptoms in constipation, assist digestion, and utilization of macronutrients, and their plausible mechanisms of action. Further, the safety of the strain will be discussed. Although the same strain has been investigated for other health benefits (13), these will not be discussed here.

## Safety

*Bifidobacterium animalis* subsp. *lactis* has been documented to be present in human food since 1980 but most likely has been consumed before that. The species is listed in Inventory of Microbial Food Cultures with Safety Demonstration in Fermented Food Products (14). The European Food Safety Authority (EFSA) has included the subspecies in the Qualified Presumption of Safety list (15). In China, *B. lactis* HN019™ has been specifically permitted for use in infant and toddler (>1 year) food since 2011 (16), and it was accepted to be Generally Recognized as Safe (GRAS, GRN445) in US in 2012 (17).

By December 2020, there were 42 studies/clinical trials published for 27 investigated cohorts, including both healthy and compromised subjects in all age groups from newborns to elderly (Table 1). The investigational products contain *B. lactis* HN019™ as a single strain or combined with other probiotics and/or prebiotics. The daily dosage of *B. lactis* HN019™ in the products ranged from 10^7^ to 10^11^ colony forming units (CFU) per day and consumption lasted for 7 days to 2 years. None of these trials have reported any safety concerns related to *B. lactis* HN019™ consumption, and it may be concluded that infants, children, adults, and seniors can safely consume *B. lactis* HN019™ at doses up to 3 × 10^11^ CFU/day.

**Table 1:** Use of *B. lactis* HN019™, including probiotic blends, in human clinical trials published until December 2020.

**Daily dose of *B. lactis* HN019™ (total probiotic potency in blend, CFU)**	**Subjects receiving products containing *B. lactis* HN019™ (N)**	**Age of subjects (median/mean, years)**	**Duration of supplementation (week)**	**Product format (delivery vehicles)**	**Other ingredients used in serum**	**Reference**
**Influence on digestive system**						
2 × 10^8^-10^9^ (8 × 10^8^-10^9^)	25 Healthy elderly	65–90 (75.3)	24	NR (powder)	*L. paracasei* Lpc-37, *L. rhamnosus* HN001, *L. acidophilus* NCFM, FOS	(18)
NR/5 × 10^9^	27 Adult constipation	19–70 (25.7)	30 days	Capsule	*L. acidophilus* NCFM, *L. casei* Lc-11, *Lactococcus lactis* Ll-23, *B. bifidum* BB-06	(19)
1–2 × 10^9^ (6–12 × 10^9^)	42 Constipated adult women	22–60 (36.84)	6	Sachet (water)	*L. rhamnosus* SP1 100MLD*, L. casei* F19, *L. acidophilus* La-14, *B. longum* BB536, *B. bacterium brief* M-16V, inulin	(20)
10^10^ (2.75 × 10^10^)	78 FC or IBS-C	18–70 (NR)	2	Capsule (drink and food)	*L. paracasei* Lpc-37, *L. acidophilus* NCFM, *B.lactis* Bl-04, *B.lactis* Bi-07	(21)
High dose: 1 × 10^10^ Low dose: 1 × 10^9^	152 Constipation	18–70 (41.7)	4	Capsule (yogurt)	NA	(22)
1 × 10^9^	14 Healthy female colleague students	>18 (NR)	2	Sachet (water or milk)	NA	(23)
5 × 10^9^	130 Healthy Preschool children	2–4 (38 months)	9 months	Capsule (milk)	NA	(24, 25)
10^9^ (2 × 10^9^)	Constipated young adults	18–45 (31.5)	2	Yogurt	Polydextrose, *L. acidophilus* NCFM	(26)
NR (2 × 10^8^-10^9^)	50 Constipation adults	18–65 (NR)	30 days	Sachet (water)	*L. paracasei* Lpc-37, *L. rhamnosus* HN001, *L. acidophilus* NCFM, FOS	(27)
1.9 × 10^7^	312 Healthy children	1–4 (21.7 months at baseline)	1 year	Reconstituted milk	GOS	(28–30)
2.17 × 10^7^-4.88 × 10^7^	80 Healthy full-term Infants	2–6 weeks (NR)	1 year	Infant formula	Gangliosides, FOS, long-chain polyunsaturated fatty acids	(31)
High dose: 1.72 × 10^10^ Low dose: 1.8 × 10^9^	66 Adult constipation	25–65 (44 in high dose, 44 in low dose)	2	Capsule (yogurt)	NA	(32)
**Modulation of immune system**						
2 × 10^8^-10^9^ (8 × 10^8^-10^9^)	25 Healthy elderly	65–90 (75.3)	24	Powder (NR)	*L. paracasei* Lpc-37, *L. rhamnosus* HN001, *L. acidophilus* NCFM, FOS	(33)^c^
1 × 10^7^	69 Pregnant women	18–35 (29.4)	8–12 weeks of gestation to the end of pregnancy	Reconstituted milk	NA	(34)
9 × 10^9^	171 Infants at risk of allergies	2–16 days at baseline (6 days)	Mothers: Pregnancy week 35 to 6 m post-partum, if breast feeding, and children: 2 years	Capsule (water, formula, breast milk, or food)	NA	(35–42)^b^
9 × 10^9^	35 Mother-baby pairs	Mother: NR Infant: 2–16 days at baseline (6 days)	Mothers: 2–5 weeks before delivery to 6 m post-partum, if breast feeding, and children: 2 years	Capsule (NR for mother, but in water, formula, breast milk, or food for infants)	NA	(43)^b^
2 × 10^10^^a^	29 Children with atopic eczema	1–10 (3.8)	12	Powder (drink or food)	*L. rhamnosus* HN001	(44)
5 × 10^9^	14 Health elderly	60–84 (69.5)	3	Sachet (milk)	NA	(45)
High dose: 5 × 10^10^ Low dose: 5 × 10^9^	30 Healthy elderly	63–84 (46)	3	Reconstituted milk	NA	(47)
5 × 10^10^	50 Healthy middle-aged subjects	41–81 (48)	3	Reconstituted milk	NA	(49)
3 × 10^11^	13 Healthy elderly	62–83 (50)	6	Reconstituted milk	NA	(51)
**Other gut health-related benefits**						
High dose: 3 × 10^9^ (2 × 10^10^) Low dose: NR (1 × 10^10^)	14 C-section-delivered neonates	0	4	NR (breastmilk)	*B. lactis* Bi-07, *L. rhamnosus* HN001, GOS	(52)
2 × 10^8^-10^9^ (8 × 10^8^-10^9^)	25 Healthy elderly	65–90 (75.3)	24	Powder (NR)	*L. paracasei* Lpc-37, *L. rhamnosus* HN001, *L. acidophilus* NCFM, FOS	(53)^c^
9 × 10^9^	171 Infants at risk of allergies	2–16 days at baseline (6 days)	Mothers: pregnancy week 35 to 6 m post-partum, if breast feeding, and children: 0–2 years	Capsule (water, formula, breast milk, or food)	NA	(54, 55) ^c^
NR (2 × 10^9^)	73 CRC patients undergone colorectal resection	NR (60.9)	7 days	Powder (water)	*L. paracasei* Lpc-37, *L. rhamnosus* HN001, *L. acidophilus* NCFM, FOS	(56)
2 × 10^9^ (8 × 10^9^)	49 CRC patients undergone surgery	NR (64.5)	5 days prior to surgery and for 14 days after surgery	Sachet (NR)	*L. paracasei* Lpc-37, *L. rhamnosus* HN001, *L. acidophilus* NCFM, FOS	(57)
High dose: 5 × 10^9^ Medium dose: 1 × 10^9^ Low dose: 6.5 × 10^7^	60 Healthy elderly	60–87 (67 in high dose, 70 in medium/dose)	4	Reconstituted milk	NA	(58)
3 × 10^10^	10 Healthy adults	20–60 (NR)	4	Reconstituted milk	NA	(59)
**Non-gut health related benefits**						
10^8^-10^10^	20 Chronic periodontitis patients	>30 (NR)	30 days	Lozenge	NA	(60)
10^9^ (4 × 10^9^)	19 Hypertensive women	34–50 (43.3)	8	Sachet (NR)	*L. paracasei* Lpc-37, *L. rhamnosus* HN001, *L.acidophilus* NCFM	(61)
2.72 × 10^10^	19 Metabolic syndrome	18–60 (48.05)	90 days	Fermented milk	NA	(62)
2.72 × 10^10^	26 Metabolic syndrome	18–60 (NR)	45 days	Fermented milk	NA	(63)

*CFU, colony forming unit; CRC, colorectal cancer; FOS, fructo-oligosaccharide; FC, functional constipation; IBS-C, constipation dominant irritable bowel syndrome; GOS, galacto-oligosaccharide; NA, not applicable; NR, not reported*.

a *The article only mentioned CFU/g, but not the g for the finished format*.

b *Studies published from the same cohort first described in Wickens et al. (36)*.

c *Studies published from the same cohort first described in de Carvalho et al. (33)*.

To date, most of the published human studies for *B. lactis* HN019™ focused on gut health of which six studies contained *B. lactis* HN019™ as a single strain product in at least one arm (Table 2).

**Table 2:** Use of *B. lactis* HN019™ as single-strain products in human clinical trials for gut health.

**Dose (CFU/day)**	**Subjects (N) in HN019 arm**	**Age (mean^a^, years)**	**Duration (week)**	**Product format**	**Endpoints**	**Reference**
High dose: 1 × 10^10^ Low dose: 1 × 10^9^	152 Adult constipation	18–70 (41.7)	4	Capsule	CTT, BMF, SC	(22)
1 × 10^9^	7 Young female with constipation	>18 (NR)	2	Sachet	BMF, SC	(23)
High dose: 1.72 × 10^10^ Low dose: 1.8 × 10^9^	66 Adult constipation	25–65 (44 in high dose, 44 in low dose)	2	Capsule	WGTT, BMF	(32)
5 × 10^9^	130 Healthy preschool children	2–4 (38 months)	9 months	Capsule	Incidence and duration of diarrhea	(24, 25)
High dose: 5 × 10^9^ Medium dose: 1 × 10^9^ Low dose: 6.5 × 10^7^	60 Healthy elderly	60–87 (67 in high dose, 70 in medium/dose)	4	Reconstituted milk	Fecal microbiota	(58)
3 × 10^10^	10 Healthy adults	20–60 (NR)	4	Reconstituted milk	Fecal microbiota	(59)

*BMF, bowel movement frequency; CFU, colony forming unit; CTT, colonic transit time; IBS-C, constipation dominant irritable bowel syndrome; CRC, colorectal cancer; FC, functional constipation; NR, not reported; SC, stool consistency; WGTT, whole gut transit time*.

a *Mean or Median*.

## Survival Through the Gut

Although not stipulated in the definition, it is often assumed that probiotic strains should be able to survive passage through the digestive system, transiently colonize in the GIT, and potentially modulate host factors, such as immune responses, digestion, or the intestinal microbiota composition and/or activity. These probiotic attributes may be observed more pronounced in subjects with suboptimal physiological status but are often not observed in the healthy subjects. These functional characterizations can be investigated in various *in vitro* and/or animal models, while ultimately, health efficacy can only be confirmed by human clinical studies (64). Although adhesion is not a pre-requisite for a strain to elicit probiotic properties, interaction with intestinal epithelial cells (IECs) and intestinal mucosa is considered important for colonization and modulation of host factors. The excellent adhesion property of *B. lactis* HN019™ to IEC lines, such as HT29, Caco-2, and HT29-MTX, were documented *in vitro* in comparison with two other probiotic *lactobacilli* strains and a negative control (non-probiotic *Lactobacillus bulgaricus*) (65).

*B. lactis* HN019™ demonstrates high tolerance to low pH and varying resistance to bile salts *in vitro*, which are two important markers for assessing survival during intestinal passage (66). So far, five human clinical studies from four cohorts investigated the survival and transient colonization of *B. lactis* HN019™ in interventions, of which only Gopal et al. (59) identified *B. lactis* HN019™ at strain level (Supplementary Table 1) (24, 25, 35, 58, 59). Regardless of the variations caused by different quantitative techniques, given the observations that *B. lactis*/bifidobacteria was/were not detected in the control group (24) or counts decreased after the cessation of the supplementation (35, 58), it is highly likely that *B. lactis*/bifidobacteria detected in the probiotic group in these studies was *B. lactis* HN019™. These studies suggest that *B. lactis* HN019™ could survive and transiently persist in intestinal transit in both short-term (2–4 weeks) and long-term (>6 months) dietary interventions in almost all age groups, including infants, toddlers, pre-school children, adults, and elderly. This, notwithstanding a high inter-individual variability (0.1–68.8%) of *B. lactis* HN019™ colonies quantified by strain-specific probes from total fecal bifidobacteria was reported in healthy adults (59). Fecal recovery has also been assessed with products containing *B. lactis* HN019™ in combination with other active components, such as prebiotics where increases in *B. lactis* or *B. lactis* HN019™ were observed (31, 34). However, Horvath et al. (67) did not observe such increases with a multi-strain probiotic containing *B. lactis* HN019™ (67).

## Modulation of Intestinal Microbiota

*Bifidobacterium* and *Lactobacillus sensu lato* have long been used in processing and preserving food and are considered as beneficial (68). Although they are not the most abundant, they are relatively stable in the adult intestinal microbiota, maintaining essential metabolic functions, such as fermentation of undigested carbohydrates into short-chain fatty acids (SCFAs), lipid metabolism, and vitamin synthesis throughout the entire lifespan (69, 70). This balance could be breached during aging, where the gut microbiota become less diverse and total bifidobacterial counts decrease (71). Therefore, sustaining these two genera at a stable level may be beneficial for maintaining a balanced healthy gut microbiota, particularly for elderly. *B. lactis* HN019™ can become a significant component of the normal fecal bifidobacterial population, and can increase total *Lactobacillus sensu lato* and *Bifidobacterium* spp. counts in feces, with daily consumption of 6.5 × 10^7^ CFU to 3 × 10^10^ CFU (58, 59). There were no significant differences between the responses of the different dose groups, indicating that even the lowest dose (6.5 × 10^7^ CFU/day) was able to confer desired changes with regards to these two beneficial bacterial groups in the intestinal microbiota (58). Furthermore, in these two studies fecal bifidobacterial and Lactobacilli counts decreased to baseline levels after cessation of the supplementation, remaining at 10^8^-10^9^ CFU/g for bifidobacteria and 10^7^-10^8^ CFU/g for lactobacilli. Interestingly, Ahmed et al. (58) reported significant increases in enterococci in the placebo group during the intervention with healthy elderly subjects. Although enterococci are normal members of the colonic microbiota, they are also opportunistic pathogens and may increase during aging (72). Moreover, in the same study, enterococci were reduced significantly in the same group during *B. lactis* HN019™ consumption, indicating that *B. lactis* HN019™ can reduce levels of fecal enterococci in elderly (58).

*B. lactis* HN019™ was shown to support maintaining the healthy/normal intestinal microbiota against aging process and by competing and excluding harmful pathogens, both at taxonomical and functional levels (25, 58, 59). In addition, further evidence for intestinal microbiota support can be found from combination products containing *B. lactis* HN019™. For example, both *B. lactis* HN019™ (10^9^ CFU/day) alone or with polydextrose (6.25 g/day) for 2 weeks increased the proportion of fecal *Bacteroides* in healthy young Japanese females, while the synbiotic format had better synergistic outcomes than probiotic alone, in terms of greater proportion of this genus among the entire microbial community (23). In a recent pilot study in Chinese neonates, after 28 days supplementation with a *B. lactis* HN019™ synbiotic since birth, the complexity and similarity of gut microbiota in cesarean-born neonates across time and across individuals were similar to those in the vaginally-born infants (52). Furthermore, both *Bifidobacterium* and *Lactobacillus* increased in their abundances after the third day of the intervention.

These findings from *B. lactis* HN019™ alone or in combination with other ingredients indicate that the above-mentioned modulations of the intestinal microbiota are ubiquitous, across age and geography.

## Maintain Intestinal Barrier Functions During Gastrointestinal Infections

Gastrointestinal infections often lead to diarrhea in humans, which was estimated as a leading cause of death among all ages across the world (73). Rotavirus, *Shigella* spp., and *Salmonella* spp. are the top three etiologies for diarrhea mortality, whereas the first two are typical for children under the age of five (73). The leading risk factors for diarrhea were unchanged from 2005 to 2015, which are poor hygiene in water supply and food chain, where bacterial pathogens are food poisoning organisms (73).

The gut forms the border between the inside (body) and the outside (lumen), an intestinal barrier is therefore indispensable and includes both a physical and an immunological barrier, preventing pathogenic bacteria and other harmful substances from entering the body while at the same time allowing nutrients and water to be absorbed. Intestinal barrier integrity is a prerequisite for homeostasis of mucosal function, which is balanced to maximize absorptive capacity, while maintaining efficient defensive reactions against chemical and microbial challenges.

### *B. lactis* HN019™ Maintains Epithelium Integrity

The inner surface of the intestine consists of a layer of cells (epithelium), which are covered by a mucus layer (a viscoelastic layer consisting mainly of protein-linked carbohydrates) which plays a key role in the barrier effect mechanism.

Tight junctions (TJ) are cell–cell junctional complexes that form a continuous intercellular barrier between epithelial cells, that the major components are claudin and occludin proteins (74). These structures control and maintain balanced intestinal permeability. Increased permeability is associated with disease conditions, which are often characterized with intestinal mucosal inflammation (74). Therefore, a proper regulation of the function of TJ is important in health maintenance and disease prevention. Putaala et al. (75) showed that cell-free supernatant (CFS) of *B. lactis* HN019™ may increase the strength of TJ, measured as trans-epithelial electrical resistance (TEER), although not to an extent that is statistically significant, while CFS of enterohemorrhagic *Escherichia coli* (EHEC) reduced the TEER significantly (75).

Cyclooxygenases (COX) play a role in normal physiological function as well as fight against pathogenic bacteria. There are two isoforms of COX in humans: COX-1, contributing to the maintenance of the physiological functions, whereas COX-2 as an inducible enzyme mediates pathogenic inflammatory responses (75). In Putaala et al. (75), CFS of both *B. lactis* HN019™ and EHEC exhibited significant increase in the ratio of COX-2 to COX-1, but the underlying mechanisms are different. EHEC CFS induced increase of COX-2 and decrease of COX-1, whereas *B. lactis* HN019™ CFS induced a slightly increase of COX-2 and retained level of COX-1, confirming the immunostimulatory properties of *B. lactis* HN019™, which lead to the activation and maintenance of normal physiological function in elderly under immunosenescence, but not pathological inflammation (47, 76).

### *B. lactis* HN019™ Shows Competitive Advantages Against Entero-Pathogens

Pre-treatment of EHEC with CFS of *B. lactis* HN019™ reduced the culturable *E. coli* numbers along with their invasive ability and cell association characteristics of the pathogenic strain (65). Both *B. lactis* HN019™ and *Salmonella typhimurium* adhere effectively to INT-407 cells, and the adhesive capacity of *B. lactis* HN019™ was similar to *S. typhimurium*. Nevertheless, the adhesion values obtained in co-treated assays showed that *B. lactis* HN019™ could significantly decrease the number of attached *S. typhimurium* while the number of attached *B. lactis* HN019™ was not affected by *S. typhimurium* (77).

### *B. lactis* HN019™ Regulates Host Immune Defense Toward Pathogens

*B. lactis* HN019™ showed potential to protect enterocytes from inflammatory responses induced by *S. typhimurium* and lipopolysaccharide (LPS) *in vitro*. A robust interleukin (IL)-8 expression was induced when INT-407 cells were exposed to *S. typhimurium*. However, live and heat-killed *B. lactis* HN019™ not only functionally modulated the epithelium by inhibiting the constitutive mRNA level of IL-8 and attenuating *S. typhimurium*-induced IL-8 gene expression, but also protect the INT-407 cells from IL-8 protein production activated by LPS. Similar results were discovered on tumor necrosis factor (TNF)-α and IL-1β gene expression. TNF-α expression was up-regulated by live *B. lactis* HN019™, but the expression levels were much lower than with *S. typhimurium* (77).

Both *S. typhimurium* and EHEC are important causes of food poisoning under poor hygienic conditions, whereas *E. coli* and rotavirus are common causes of diarrhea in infants and young animals. The latter two infectious agents also commonly cause diarrhea in piglets during weaning, making piglets an ideal model for studying this type of gastrointestinal infections. The administration of *B. lactis* HN019™ to weaning piglets resulted in a significantly lower incidence of diarrhea during the first 2 days after weaning. The fecal levels of *E. coli* and rotavirus were also lower in the treatment group, which exhibited a significantly higher titer of specific antibodies in the feces (78). In addition, in mice models, *B. lactis* HN019™ was found to protect the animals against *S. typhimurium* and EHEC with lower rate of morbidity (79, 80). The probiotic-fed mice also showed significantly higher numbers of phagocytic active leukocytes (80). These findings indicated a mechanism of enhanced immune-mediated protection by *B. lactis* HN019™ against gastrointestinal infections. Moreover, in a large randomized trial in India, *B. lactis* HN019™ showed efficacy in reducing the incidence of diarrhea and fever during the rainy season (months August and September), when incidence of diarrhea is the highest (24). Fecal immunoglobulin (Ig)A and serum inflammatory marker IL-8 were also significantly decreased in the *B. lactis* HN019™ arm compared to placebo (24). Similar observations were made with *B. lactis* HN019™ in combination with galacto-oligosaccharides (GOS); reducing the incidence of dysentery and showing a trend (*p* = 0.08) for reduced incidence of diarrhea (28).

## Regulate Gut Motility and Improve Symptoms in Constipation

Constipation without identifiable organic causes, may refer to slow-transit constipation (STC) or obstruction constipation, which are related to poor lifestyles, such as high fat/protein diet and lack of fiber and liquid intake and exercise. The typical clinical symptom of STC is the prolonged gut transit time (GTT), which may be resulted from the dysfunction of colonic smooth muscles (81).

*B. lactis* HN019™ showed prokinetic effects on subjects suffered functional constipation (FC). For example, daily intake of *B. lactis* HN019™ at both high (1.72 × 10^10^ CFU) and low (1.8 × 10^9^ CFU) doses for 2 weeks decreased colonic transit time (CTT) in 100 constipated adults, with no change observed in the placebo group (32). This finding of shorten CTT was not observed in Ibarra et al. (22), who investigated the effects of *B. lactis* HN019™ using a similar intervention regime but longer duration (4 weeks) in 228 constipated adults diagnosed with the same ROME III criteria as in Waller et al. (32). However, a sub-group of subjects (*N* = 65) with bowel movement frequency (BMF) of less than three bowel movements per week (i.e., those who were constipated) benefitted from the consumption of the strain (22). After 4 weeks, daily doses of 10^10^ CFU (high does) and 10^9^ CFU (low dose) increased 2 and 1.7 bowel movements/week, respectively. The stool consistency outcomes indicated that the participants in Ibarra et al. (22) were mild-constipated subjects (Bristol Stool Scale: 3.3) without changing their level of constipation after the intervention. However, Aoe et al. (23) did not observe an effect of *B. lactis* HN019™ alone on BMF in constipated women after 2-week supplementation. The study population was, however, under-powered; with only seven subjects per treatment group (23). In the same study, Aoe et al. (23) explored the outcomes on stool characteristics in a synbiotic intervention arm, where polydextrose (6.25 g/day) and *B. lactis* HN019™ (10^9^ CFU/day) were combined. This synbiotic showed increased stool amounts which positively correlated with the relative abundance of *Bacteroides*. It is important to note that PDX has been reported to positively influence stool consistency and bowel function (82). Other studies with *B. lactis* HN019™ in combination with other probiotics or prebiotics observed improvements after intervention in bowel functions, such as shortened CTT (26), increased stool frequency and consistency (18, 27), reduced flatulence symptoms (21). However, although Botelho et al. observed a change in fecal microbiota composition, the study failed to observe a positive effect on bowel function as compared to the placebo group (19) (Table 1).

**Figure 1:**
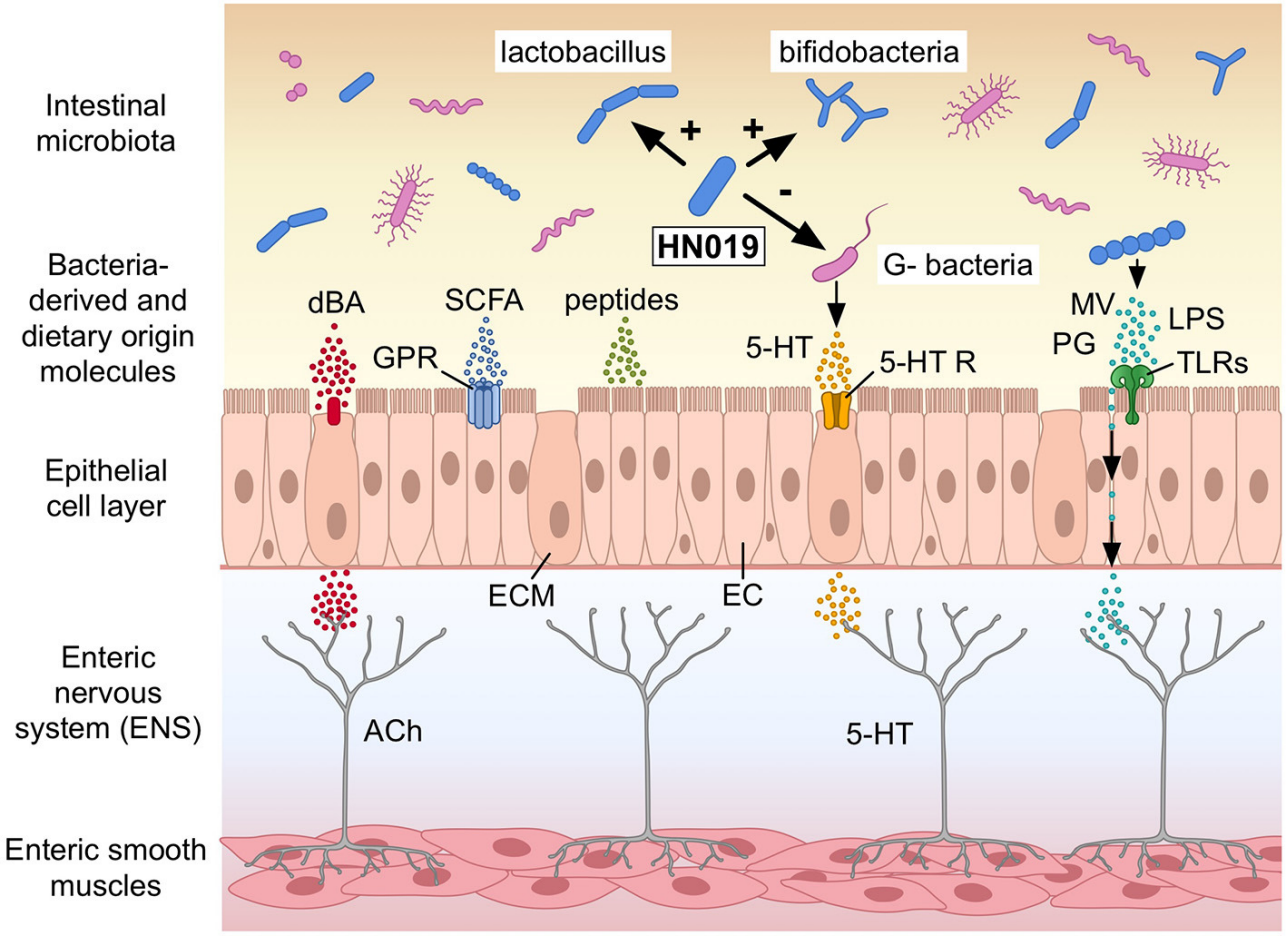
Potential mechanisms how *Bifidobacterium lactis* HN019™ (HN019) may modulate colonic motility in humans. The human intestinal epithelial cell layer comprises, among others, of epithelial cells (EC) and enterochromaffin cells (ECM) lining the gut wall. *B. lactis* HN019™ may stimulate Lactobacilli and Bifidobacteria and inhibit certain Gram-negative bacteria (G^−^ bacteria). Bacteria-derived and neurotransmitters or modulators of dietary origin, such as deconjugated bile acids (dBA), short chain fatty acids (SCFA), and serotonin (5-HT) could interact with their host receptors expressed in the epithelial cell layer; G protein-coupled receptors (GPR); and 5-HT receptors (5-HT R). Microvesicles (MV), peptidoglycan (PG), and lipopolysaccharide (LPS) from different bacteria interact with Toll-like receptors (TLRs). These components may also cross the epithelial layer and like the receptors signal afferent neurons in the enteric nervous system (ENS) with among others acetylcholine (ACh) to regulate colonic motility. Modified after Dalziel et al. (2021) (with permission). ©Pinja Kettunen/SciArt and IFF, with permission.

These observations on bowel habits led to the hypothesis that *B. lactis* HN019™ affects colonic motility patterns, which most probably via direct interactions with host factors rather than alteration of water and electrolyte secretion, even though improvements for stool amount were observed in combination with polydextrose, which is a soluble fiber. This hypothesis was investigated in an *ex vivo* model, where *B. lactis* HN019™ extract markedly increased the contractile amplitude of synchronous contractions spanning the proximal colon to the rectum. Interestingly, this effect occurred post-treatment (83). Even though the exact components of *B. lactis* HN019™ extract are not clear, several potential mechanistic pathways including potential active molecules will be reviewed below (Figure 1).

### Modulate Microbiota–Fermentation–Gut–Brain Signaling

The primary pathophysiological mechanism for constipation is gut dysmotility, presenting dysregulated or deficient colonic propulsive motor patterns (6, 84). So far, molecules reported to impact on gut motility are SCFAs, formylated peptides, and serotonin (5-hydroxytryptamine, 5-HT), which might be derived from bacterial surface structures or bacterial fermentation of nutrients (6, 85). Short-chain fatty acid concentrations can directly influence motility through the G protein-coupled receptor (GPR) 41 and GPR43, where the underlying mechanisms have been mostly addressed in animal studies (86–88). For example, intra-luminal administration of a blend of acetate, propionate and butyrate in rat was shown to lead to an increased 5-HT concentration and subsequently decreased CTT (86). *B. lactis* HN019™ has been shown to produce lactate and acetate, where acetate was more selective potent agonist for GPR43 (48, 89). However, there are a few studies confirming the effects of SCFAs on motility in humans (90, 91).

In addition, *B. lactis* HN019™ may also influence gut motility via gut commensals, which are known to be involved in serotonin biosynthesis and bile acid metabolism (92, 93). Bile salts are known to stimulate colonic contractility and transit in humans (94–96), where probiotic strains may deconjugate bile salts, leading to the formation of secondary bile salts with laxative effects (6). However, to date, no data is available on the ability of *B. lactis* HN019™ to deconjugate bile salts.

### Improve Gastrointestinal Symptoms in Constipation

Except bowel habits, *B. lactis* HN019™ has been reported to reduce the frequency of functional gastrointestinal symptoms in adults, including vomiting, regurgitation, abdominal pain, nausea, gurgling, constipation, diarrhea, and flatulence (32). Most symptoms improved in the high dose (eight of nine symptoms) and low dose (seven of nine symptoms) groups, respectively, while only two of nine symptoms showed a statistically significant improvement with placebo. In Ibarra et al. (22), when comparing the measured gastrointestinal symptoms between subjects taken *B. lactis* HN019™ vs. placebo, only a reduction observed in the degree of straining in the sub-group of subjects (BMF<3). Improvements of gastrointestinal symptoms were also observed in studies with *B. lactis* HN019™ in combination with other probiotics or prebiotics (21, 27) (Table 1).

## Assist Digestion and Utilization of Macronutrients

Some of the constipation related symptoms are associated with inadequate digestion of fibrous components from the diet. Plant-based diets, rich in fermentable residues, could be a solution to constipation but may be accompanied with complaints of gas produced during endogenous microbial fermentation. Aiding their digestion may thus help reduce these symptoms. *In vitro, B. lactis* HN019™ was shown to utilize commercial oligosaccharides: FOS, GOS, and xylo-oligosaccharide (XOS) (46, 50). Further, the analysis of complete genomes from several commercial *B. lactis* strains, including *B. lactis* HN019™ supports this, as well as the identification of several putative carbohydrate-modifying enzymes in the genome of *B. lactis* HN019™ for a wide range of complex carbohydrates (97, 98). This is supported by the utilization of oligosaccharides by *B. lactis* HN019™ seems best for mono- and di-saccharides, such as stachyose, raffinose, but not able to utilize oligosaccharides with a degree of polymerization of more than 7 (99, 100). Such oligosaccharide utilization may contribute to a reduced fermentation by the endogenous microbiota and improve tolerance to such fibers. *Bifidobacterium* spp. are usually non-gas producers, since in general, they metabolize monosaccharides via the fructose-6-phosphate pathway eventually to SCFAs without gas as a by-product (98, 101). Therefore, endogenous and consumed bifidobacteria, including *B. lactis* HN019™, could divert the fermentation in the colon toward non-gaseous end-products. This property of *B. lactis* HN019™ may support tolerance for the fermentation of oligosaccharides *in vivo*, which is in line with clinical observations of *B. lactis* HN019™ on reduced flatulence (21, 32). In addition, lactate and acetate are produced during *B. lactis* HN019™ fermentation, indicating potential roles in digestion of dietary components without differentiation of the origin; carbohydrate or protein (89, 102).

Weak indications were observed for the involvement of *B. lactis* HN019™ in digestion of specific dietary component(s) in human (44). In this study, a combination of *L. rhamnosus* HN001 and *B. lactis* HN019™ was examined on established atopic dermatitis (AD) in children SCORAD (SCORing Atopic Dermatitis), a measure of the extent and severity of AD, was assessed at baseline, 2 and 12 weeks after starting treatment and 4 weeks after treatment was discontinued. The supplement alleviated AD symptoms, but only in a sub-group of food-sensitized children, whereas no effect on children sensitized to environmental allergens, suggesting the beneficial effect of the probiotic may only relate to local effects on GIT, mainly toward food challenges (44). However, since the results came from sub-group analysis in children only tested for several common food allergens, the results should be interpreted with caution, and more confirmatory human studies should be carried out in larger population.

Gut microbial bile salt hydrolase (BSH) enzymes promote deconjugation, dehydrogenation, and dihydroxylation of primary bile acids, increasing the chemical diversity of bile acids, which can in turn have an impact on host lipid metabolism (93). Unconjugated bile acids are less efficient emulsifiers of dietary fats and may positively influence blood lipid profiles. Even though no data showed *B. lactis* HN019™ could deconjugate bile salts, as discussed earlier, *B. lactis* HN019™ may have a role in bile acid metabolism via affecting gut commensals, which in turn can change the amount of fat that the body is able to absorb. In addition, several studies have shown the potential of *B. lactis* HN019™ alone or in combination with other probiotics in improving blood lipid profiles (61, 63).

## Discussion

Different aspects of digestive health are the most common health benefits addressed by probiotics (103). The above review has shown that also *B. lactis* HN019™ has several health benefits in this area, in particular in the area of bowel function and intestinal motility. *Bifidobacterium animalis* subsp. *lactis* is one of the most common lactic acid producing probiotics in North-America and Europe (104). Its superiority in the dietary supplement and dairy industry has yielded several well-known commercial strains, such as *B. lactis* DN-173 010/*B. lactis* CNCM I-2494 (Danone), BB-12 (Chr. Hansen), and the here discussed *B. lactis* HN019™ (International Flavors & Fragrances).

*B. lactis* DN-173010/*B. lactis* CNCM I-2494 in fermented milk at a dosage of 1.25 × 10^10^ CFU two to three times/day, has shown beneficial effects on acceleration of gastrointestinal transit in both a healthy and constipation dominant irritable bowel syndrome (IBS-C) population after 10 days to 4 weeks intervention (105–107). Moreover, this probiotic fermented milk (PFM) has been shown to improve general gastrointestinal well-being, reduce symptomatology including abdominal distention and discomfort in individuals from the general population with minor digestive symptoms but not functional gastrointestinal disorders (107–109), as well as in IBS-C population (106). These studies were all performed with fermented milk which also contained the classic yogurt cultures: *S. thermophilus* and *L. bulgaricus*. Noteworthy, both Guyonnet et al. (107) and Marteau et al. (109) recruited healthy women without any diagnosis of digestive disease, including FBD, such as IBS with normal BMF (3–21/week). This hypothesis was further confirmed, in rat model, where *B. lactis* CNCM I-2494 reduced stress-induced visceral hypersensitivity by restoring intestinal barrier function (110), and in a proof-of-concept study where healthy women consumed this PFM and modulation of brain activities related to mood was reported (111).

*B. lactis* BB-12 is another well-documented probiotic strain that has been investigated for its digestive health benefits (112). In several studies the strain has been evaluated for the effects it has on BMF. In a large study testing two doses; 10^9^ and 10^10^ CFU/day for four weeks, a beneficial effect was observed especially in the lower tested dose (113). Similarly, a fermented oat product with *B. lactis* BB-12 was observed to normalize bowel function in elderly. In combination with *Lacticaseibacillus paracasei* CRL-341 softer stools were observed (114). This could suggest a potential mechanism of action. A recent study was, however, not able to replicate the effect on intestinal transit time (115); most likely because of the small number of subjects and that subjects were not selected to have long transit times or be constipated. Consumption of yogurt or a capsule with the strain was not found to change the intestinal microbiota, this is not unusual; the resilience of a healthy colonic microbiota is likely to resist change from one (probiotic) strain (116). However, consumption of the probiotic yogurt did lead to fecal recovery of the strain (115).

In summary, all three *B. lactis* strains discussed above do seem to have quite similar effects on bowel movements. This is confirmed by a recent meta-analysis which concluded that *B. lactis* may be more effective in increasing stool frequency and in improving stool consistency (117). It is therefore tempting to speculate that this relates to the high genome similarity and almost complete synteny of the genomes between various strains within the subspecies *B. lactis* (97, 118). The small genetic difference between strains of *B. lactis* could be investigated further and correlated with phenotypic traits to help understand mechanisms of action.

As manifested by the current research, serotonin signaling pathway seems to be a major effector pathway underlining the efficacy of *B. lactis* HN019™ on gut motility, where SCFAs derived from gut microbial fermentation seem to be promising candidate effector molecules. The contribution from other bacterial derived molecules, such as formylated peptides acting on FPR1 cannot be ruled out with the current knowledge. Although probiotics in healthy subject generally cause no or minor changes in the gut microbiota (113); it nevertheless seems to be involved in the complex interactions of *B. lactis* HN019™ with host metabolisms and immune defenses, including endocrine cells, immune cytokines, and neurotransmitters, which are yet to be fully understood. Therefore, future investigations on the exact role of *B. lactis* HN019™ on gut motility should focus on identifying key molecules and signaling pathways in the gut–brain–microbiota interactions in humans, which could enhance our understanding. To this end, focus of microbiota analysis should shift or expand to the analysis of the colonic mucosal microbiota and metabolomics of the intestinal microbiota (119). Recent scientific advances suggest great potential for bacterial-derived microvesicles (MVs) on gut health. Microvesicles from *L. reuteri* DSM 17938 were shown in an *ex vivo* mouse colon to recapitulate the effects of the whole bacteria on increasing colonic propagating contraction frequency (120). Application of *L. rhamnosus* JB-1 and its MVs separately to the apical side of the gut epithelium, could reduce the amplitude of propagating neural contractions in *ex vivo* mouse colon via interactions with ENS (121). These findings suggest a role for MVs from probiotics on signaling and their significance in the mechanisms of action within the gut–brain–microbiota axis, would therefore be potential mode of action to be investigated in the future for a better understanding of mechanisms of *B. lactis* HN019™.

In conclusion, *B. lactis* HN019™ has been observed to have benefits on different aspects of digestive health; not unlike some other commercial probiotic *B. lactis* strains. What is needed, however, is a better understanding of the mechanisms of action on how *B. lactis* HN019™ and probiotics in general function (122). Further, clinical trials should be better designed; taking into account the heterogeneity across different populations (123, 124) with sufficiently sized studies and clearly defined endpoints.

## Author Contributions

JC, AL, and AO: conceptualization, review, and editing. JC and AO: original draft preparation. All authors have read and agreed to the published version of the manuscript.

## Conflict of Interest

At the time of writing, the authors were employed by International Flavors & Fragrances Inc. (IFF). IFF manufactures and markets *Bifidobacterium animalis* subsp. *lactis* HN019.

## Publisher's Note

All claims expressed in this article are solely those of the authors and do not necessarily represent those of their affiliated organizations, or those of the publisher, the editors and the reviewers. Any product that may be evaluated in this article, or claim that may be made by its manufacturer, is not guaranteed or endorsed by the publisher.
