# Supplementary material for: Bifidobacterium animalis subsp. lactis HN019 Effects on Gut Health: A Review
Source: Front Nutr. 2021 Dec 14;8:790561. doi: 10.3389/fnut.2021.790561 (PMC8712437; doi:10.3389/fnut.2021.790561)
Supplement: Supplementary file 1 [file Table_1.docx]

Supplementary Material

# Supplementary Tables

**Table S1.** Effects of supplementation of *B. lactis* HN019™ alone on intestinal bacterial groups

| Daily dose of  *B. lactis* HN019™ (CFU) and Duration of supplementation | Subjects receiving products containing *B. lactis* HN019™ (N) | Age of subjects (median/mean, years) | Technics used to assess intestinal microbiota | Effects on intestinal microbiota | Reference |
| --- | --- | --- | --- | --- | --- |
| 9×10^9^  mothers (from 35 weeks gestation until 6 months post-partum if breastfeeding) and infants (from birth to 2 years) received intervention | 50 samples without eczema or early antibiotic use  Infants at risk for allergic diseases, such as asthma, eczema and hay fever | Median age of 6 days after birth for infants to start supplementation | Metagenomics | - *B. lactis* HN019™ supplementation does not have any effects on clinical endpoints  - *B. lactis*: observed in all arms, but significantly more abundant in *B. lactis* HN019™ arm at all timepoints (birth, 3 months, 12 months and 24 months) (readily detected in the stool when administered in early life, but did not otherwise alter the diversity or overall composition of the gut microbiota in these children). | (Murphy et al., 2019) |
| 5x10^9^  9 months | 130  Healthy preschool children | 2-4 (38 months) | bifidobacteria/lactobacilli: cultivation on Beeren’s medium | -bifidobacteria/lactobacilli: no significant difference in treatment vs. control  *-B. lactis* was not observed in placebo via culturing/qPCR, but can be recovered live in most of the children consumed *B. lactis* HN019™ and detected in all treated subjects via qPCR  -*B. lactis* was also significantly higher in the arm supplemented with *B. lactis* HN019™ at the end of 6 months | (Hemalatha et al., 2014; Hemalatha et al., 2017) |
|  |  |  | *Bifidobacterium spp.:* qPCR |  |  |
|  |  |  | *B. lactis*: qPCR |  |  |
|  |  |  | *B. lactis* HN019™: not measured |  |  |
| High dose: 5x10^9^  Medium dose: 1x10^9^  Low dose: 6.5x10^7^  4 weeks | 60  Healthy elderly | 60-87 (67 in high dose, 70 in medium/dose) | bifidobacteria/lactobacilli: cultivation on Beeren’s medium | -bifidobacteria significantly increased in all three tested arms during supplementation but decreased in post-intervention period. However, even bifidobacteria in placebo remain above 10^9 CFU/g throughout the entire intervention.  -lactobacilli: a trend of increase during supplementation in all three tested arms  -enterobacteria: increased in placebo (aging effects), and significantly reduced in the same tested arm during supplementation (colonization resistance against entero-pathogenic bacteria in elderly). | (Ahmed et al., 2007) |
|  |  |  | *B. lactis*: not measured |  |  |
|  |  |  | *B. lactis* HN019™: not measured |  |  |
| 3x10^10^  4 weeks | 10  Healthy adults | 20-60 (NR) | bifidobacteria: cultivation on Beeren’s medium | - bifidobacteria: average increase of 0.42 log units after 4 weeks supplementation (p<0.05) (log10, 8.78 to 9.2). Counts returned to the level before supplementation after cessation the consumption of *B. lactis* HN019™ (log 10, 8.6). CFU/g.  -lactobacilli: significant increase of 1.33 log units after 4 weeks supplementation (p<0.05). Counts returned to the level before supplementation after cessation the consumption of *B. lactis* HN019™.  -A trend of decreased of Enterobacteria during supplementation.  -No change of any measured taxa in placebo group.  - *B. lactis* HN019™ / bifidobacteria: average 28% (0.1%-68.8%)  -No observed *B. lactis* HN019™ before supplementation | (Gopal et al., 2003) |

Ahmed M, Prasad J, Gill H, Stevenson L, Gopal P. Impact of consumption of different levels of *Bifidobacterium lactis* HN019 on the intestinal microflora of elderly human subjects. *J Nutr Health Aging*. (2007) 11:26–31

Gopal PK, Prasad J, Gill HS. Effects of the consumption of *Bifidobacterium lactis* HN019 (DR10 TM) and galacto-oligosaccharides on the microflora of the gastrointestinal tract in human subjects. *Nutr Res.* (2003) 23:1313–28. doi: 10.1016/S0271-5317(03)00134-9

Hemalatha R, Ouwehand AC, Forssten SD, Geddan JB, Mamidi RS, Bhaskar V, et al. A community-based randomized double blind controlled trial of *Lactobacillus paracasei* and *Bifidobacterium lactis* on reducing risk for diarrhea and fever in preschool children in an urban slum in India. *Eur J Food Res Rev*. (2014) 4:325–41. doi: 10.9734/EJNFS/2014/8280

Hemalatha R, Ouwehand AC, Saarinen MT, Prasad UV, Swetha K, Bhaskar V. Effect of probiotic supplementation on total Lactobacilli, bifidobacteria and short chain fatty acids in 2-5-year-old children. *Microb Ecol Health Dis.* (2017) 28:1298340. doi:10.1080/16512235.2017.1298340

Murphy R, Morgan XC, Wang XY, Wickens K, Purdie G, Fitzharris P, et al. Eczema-protective probiotic alters infant gut microbiome functional capacity but not composition: sub-sample analysis from a RCT. Benef Microbes. (2019) 10:5–17. doi: 10.3920/BM2017.0191
